# Low cancer yield in PI-RADS 3 upgraded to 4 by dynamic contrast-enhanced MRI: is it time to reconsider scoring categorization?

**DOI:** 10.1007/s00330-023-09605-0

**Published:** 2023-04-13

**Authors:** Emanuele Messina, Martina Pecoraro, Ludovica Laschena, Marco Bicchetti, Flavia Proietti, Antonio Ciardi, Costantino Leonardo, Alessandro Sciarra, Rossano Girometti, Carlo Catalano, Valeria Panebianco

**Affiliations:** 1 Department of Radiological Sciences, Oncology and Pathology, Sapienza University/Policlinico Umberto I, Viale del Policlinico 155, 00185 Rome, Italy; 2 Department of Maternal-Infant and Urological Sciences, Sapienza University/Policlinico Umberto I, Viale del Policlinico 155, 00185 Rome, Italy; 3 Institute of Radiology, Department of Medicine, University of Udine, University Hospital S, Maria Della Misericordia; P.Le S. Maria Della Misericordia 15, 33100 Udine, Italy

**Keywords:** Multiparametric magnetic resonance imaging, Prostatic neoplasms, Biopsy

## Abstract

**Objectives:**

To evaluate MRI diagnostic performance in detecting clinically significant prostate cancer (csPCa) in peripheral-zone PI-RADS 4 lesions, comparing those with clearly restricted diffusion (DWI-score 4), and those with equivocal diffusion pattern (DWI-score 3) and positive dynamic contrast-enhanced (DCE) MRI.

**Methods:**

This observational prospective study enrolled 389 men referred to MRI and, if positive (PI-RADS 3 with PSA-density [PSAD] ≥ 0.15 ng/mL/mL, 4 and 5), to MRI-directed biopsy. Lesions with DWI-score 3 and positive DCE were classified as “PI-RADS 3up,” instead of PI-RADS 4. Univariable and multivariable analyses were implemented to determine features correlated to csPCa detection.

**Results:**

Prevalence of csPCa was 14.5% and 53.3% in PI-RADS categories 3up and 4, respectively (*p* < 0.001). MRI showed a sensitivity of 100.0%, specificity 40.9%, PPV 46.5%, NPV 100.0%, and accuracy 60.9% for csPCa detection. Modifying the threshold to consider MRI positive and to indicate biopsy (same as previously described, but PI-RADS 3up only when associated with elevated PSAD), the sensitivity changed to 93.9%, specificity 57.2%, PPV 53.0%, NPV 94.8%, and accuracy 69.7%. Age (*p* < 0.001), PSAD (*p* < 0.001), positive DWI (*p* < 0.001), and PI-RADS score (*p* = 0.04) resulted in independent predictors of csPCa.

**Conclusions:**

Most cases of PI-RADS 3up were false-positives, suggesting that upgrading peripheral lesions with DWI-score 3 to PI-RADS 4 because of positive DCE has a detrimental effect on MRI accuracy, decreasing the true prevalence of csPCa in the PI-RADS 4 category. PI-RADS 3up should not be upgraded and directed to biopsy only if associated with increased PSAD.

**Key Points:**

• *As per PI-RADS v2.1 recommendations, in case of a peripheral zone lesion with equivocal diffusion-weighted imaging (DWI score 3), but positive dynamic contrast-enhanced (DCE) MRI, the overall PI-RADS score should be upgraded to 4*.

•* The current PI-RADS recommendation of upgrading PI-RADS 3 lesions of the peripheral zone to PI-RADS 4 because of positive DCE decreased clinically significant prostate cancer detection rate in our series.*

• *According to our results, the most accurate threshold for setting indication to prostate biopsy is PI-RADS 3 or PI-RADS 3 with positive DCE both associated with increased PSA density.*

**Supplementary Information:**

The online version contains supplementary material available at 10.1007/s00330-023-09605-0.

## Introduction

Over the last few years, evidence has established magnetic resonance imaging (MRI) as the most accurate and cost-effective diagnostic imaging modality for the detection and staging of clinically significant disease (csPCa) [1–4]. Recent trials validated the so-called "MRI pathway" as a mean to trigger MRI-informed biopsy strategies in biopsy-naïve patients [5–9], increasing the detection of csPCa, linked to worst outcomes, while allowing a reduction of overdiagnosis of clinically insignificant prostate cancer (ciPCa), which do not cause symptoms nor death [10]. However, the positive predictive value (PPV) of MRI for csPCa detection is still low and widely variable (35% for PI-RADS score ≥ 3 and 49% when ≥ 4) [11], meaning that many suspicious MRI findings are in fact false-positives on targeted biopsy.

Currently, international guidelines recommend performing MRI, acquired and reported using the Prostate Imaging—Reporting and Data System (PI-RADS) v2.1 [12–14]. PI-RADS recommendations suggest performing MRI-directed biopsies (MRDB) on lesions classified as PI-RADS scores 4 and 5, while the management of PI-RADS 3 findings is still currently highly debated. Indeed, this category is one of the main sources of false-positive biopsy results [15]. The cancer yield of findings classified as PI-RADS 3 is still uncertain, with a prevalence of csPCa in biopsied cases spanning from 3 to 50% in the literature [15, 16]. As per PI-RADS v2.1 recommendations, a lesion without positive dynamic contrast-enhanced (DCE) MRI should be classified as PI-RADS 3 when showing an equivocal appearance on diffusion-weighted imaging (DWI) and apparent diffusion coefficient (ADC) map (DWI score 3) (Supplementary Table 1). In case of lesions with equivocal DWI/ADC (DWI score 3), but positive DCE, the overall PI-RADS score should be upgraded to 4 [17]. This upgrade must be considered only in case of peripheral zone (PZ) lesions, which represent the vast majority of prostate lesions and are associated with poorer clinical outcomes [17–19].

On this basis, there are two possible types of PI-RADS 4 assignments: those with clearly restricted diffusion (DWI score 4) and size lower than 1.5 cm, and those deriving from the upgrading of a peripheral zone PI-RADS 3 lesion showing positive DCE. As far as we know, no previous prospective works investigated the prevalence of csPCa in PI-RADS 4 lesions deriving from PI-RADS 3 upgrading. Based on our empirical experience in a tertiary referral high-volume center, many of those lesions result in negative or ciPCa on targeted biopsy.

Hence, the primary aim of the study was to evaluate the diagnostic performance of MRI and PI-RADS score in detecting csPCa in PI-RADS 4 categories with clearly restricted diffusion (DWI score 4) or after upgrading from PI-RADS 3 category because of positive DCE and assess the impact of a revised PI-RADS categorization on biopsy decisions. As a secondary objective, we aimed at determining the clinical and radiological features associated with csPCa in those subcategories.

## Materials and methods

### Patient population and study design

**Fig. 1:**
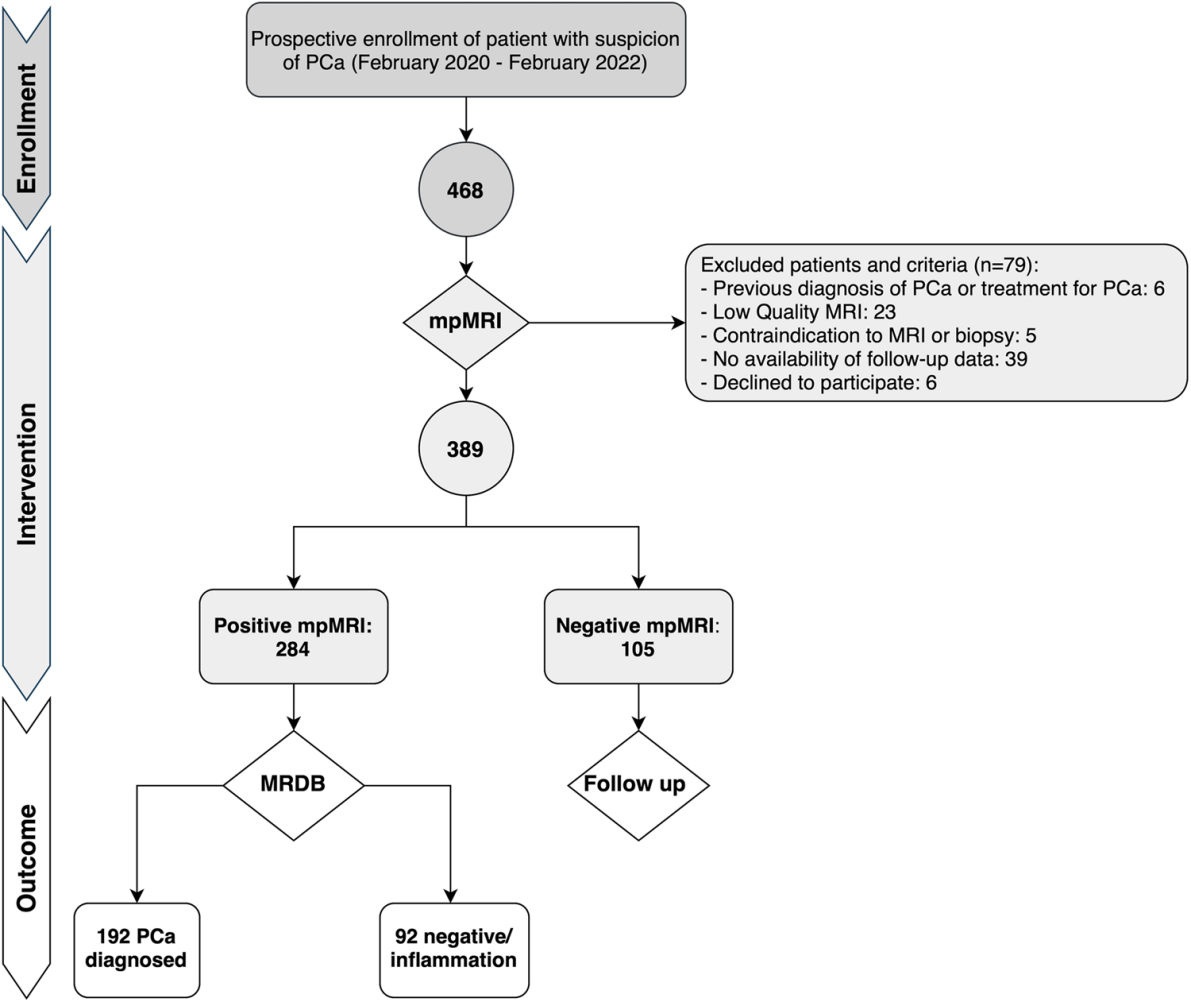
Study flowchart showing the outline of its different phases: enrollment, intervention, and outcomes. PCa, prostate cancer; MRI, magnetic resonance imaging; MRDB, MRI-directed biopsy

This observational prospective single-center cohort study received formal Institutional Review Board and Ethical Committee approval. Written informed consent was obtained from each participant. A total cohort of 468 patients was consecutively enrolled from February 2020 to February 2022. According to the study design, men with clinical suspicion of PCa (total PSA > 3.0 ng/mL, or > 2.5 in patients with family history, and/or positive digital rectal examination [DRE], and/or suspicious findings at ultrasound) were referred to MRI and, if positive to MRDB (see below for the definition of “positive”). Inclusion criteria also included an acceptable MRI quality to rule in and/or rule out csPCa; only MR images with a Prostate Imaging Quality score [20] (PI-QUAL) ≥ 3 were included. Exclusion criteria were a prior diagnosis of PCa, previous prostate biopsy, dementia or altered mental status, and any contraindication to MRI or biopsy procedures (Fig. 1).

### MRI protocol, image interpretation, and MRDB

All exams were conducted on two 3 Tesla MRI Scanners (GE Discovery 750 and MAGNETOM Vida Siemens), using a 32-channel surface phased-array body coil. According to PI-RADS v2.1 recommendations, all the exams were acquired using a multiparametric protocol, which included high-resolution T2-weighted imaging (T2WI) on the axial and coronal planes, DWI at b values of 100, 800, 1000, and 2000s/mm^2^ (the last as part of a separate acquisition) with ADC map reconstruction (based on 100, 800 and 1000 s/mm^2^), and perfusion (DCE) at a temporal resolution of 6 s following an intravenous bolus of gadolinium-based contrast media. MRI acquisition parameters are listed in Supplementary Table 2.

Images were reported independently by two readers both qualifiable as experts according to the European Society of Urogenital Radiology (ESUR) / EAU Section of Urologic Imaging (ESUI) consensus criteria (15 and 5 years of experience in prostate imaging, who are referred to respectively as “more” and “less experienced reader” throughout the text) [21, 22]. For image interpretation, readers used adjusted PI-RADS v2.1 rules for the PZ as follows: (i) when a lesion showed equivocal diffusion pattern (DWI score 3) and negative DCE, the lesion was categorized as PI-RADS 3; (ii) when a PI-RADS 3 lesion (DWI score 3) was associated to positive DCE, that lesion was categorized as “PI-RADS 3up” rather than as PI-RADS 4. Therefore, PI-RADS 4 categorization included only those lesions showing clearly restricted diffusion (DWI score 4). Readers also attributed the PI-QUAL score to each MRI examination. For images acquired before the introduction of the quality scoring system in 2020, readers retrospectively excluded PI-QUAL < 3 MR images.

MRDBs were performed by the same team of radiologists, with a targeted technique. Cores were obtained with a transrectal approach using a dedicated system (Urostation Koelis) [23]. The selected MRI threshold for setting indication to biopsy was PI-RADS 3 associated with PSA density ≥ 0.15 ng/mL/mL; therefore, all PI-RADS 3up lesions were directed to MRDB.

A dedicated pathologist with 15 years of experience in genitourinary pathology reported the biopsy cores according to the International Society of Urological Pathology (ISUP) grading groups, after they were stored in formalin-filled containers.

### Standard of reference and statistical analysis

For the purpose of the study, analysis was performed on per-index lesion basis. Index lesion was defined as the MRI finding showing the higher PI-RADS category or, being equal to the category, the one showing the largest size or extra-prostatic extension. After matching MRI images with biopsy results, MRI findings were assessed as true-positive if matching with the presence of csPCa, and false-positive if not (no cancer or ciPCa). True-negative findings correspond either to a negative histopathologic finding at biopsy or to a nonprogressive, negative follow-up MRI after at least 12 months, without an increase of serum PSA value. Only patients with at least one proper follow-up examination were included (Fig. 1).

Data analysis focused on two different outcomes, namely “outcome 1”, standing for the identification of overall PCa (both ciPCa and csPCa), and “outcome 2”, standing for the detection of csPCa only. The latter was defined as ISUP grading group ≥ 2 [12]. The prevalence of csPCa and ciPCa was calculated for all the different PI-RADS categories, including for PI-RADS 3up. The performance of MRI was assessed by means of receiver operating characteristic (ROC) analysis, according to both readers’ assessments for both outcomes, deriving sensitivity, specificity, PPV, NPV, and accuracy according to the Youden index. For both outcomes, analysis was performed using two different thresholds to define MRI positive, determining biopsy indication: (i) PI-RADS 3 category with increased PSAD, and (ii) an adjusted threshold using PI-RADS 3 or 3up category with increased PSAD.

Inter-reader agreement analysis was performed with Cohen’s kappa (k) statistics for overall PI-RADS assessment and on a per-sequence basis. Differences between patient demographics, clinical characteristics, and PI-RADS score were analyzed using the analysis of variance (ANOVA). Univariable and multivariable analysis with logistic regression was performed to assess which of the following variables was independently predictive of csPCa: patient age (< 70 vs ≥ 70), family history (positive vs negative), PSA value (< 10.0 vs > 10.0 and < 20.0 vs > 20.0 ng/mL), PSA density value (< 0.15 vs ≥ 0.15 ng/mL/mL), the total number of MRI suspicious foci [1 vs > 1], best MRI sequence on which the finding is identifiable (DWI/ADC vs DCE) and the PI-RADS score.

Statistical analysis was performed using Statistical Package for the Social Sciences (SPSS) v28.0.1.1. Tests were two-sided, and statistical significance was set at *p* < 0.05.

## Results

### Patient characteristics

The 389 patients included in the final analysis presented a median age of 67.2 years (interquartile range [IQR] 43–85), median total PSA value of 8.42 ng/mL (IQR 0.08–86.20), and median PSA density of 0.19 ng/mL/mL (IQR 0.002–1.60), with a statistically significant difference in PSA density between patients without PCa or having ciPCa and csPCa (0.13 vs 0.16 vs 0.3, *p* < 0.001). The clinical, radiologic, and pathologic characteristics of the final cohort are summarized in Table 1.

**Table 1:** Summary of cohort population’s clinical, radiological, and pathological data

Variable	Total cohort	PCa	csPCa	ciPCa	Negative	*p* value*
Sample Size, *n (%)*	389 (100)	192 (49.4)	132 (33.9)	60 (15.4)	197 (50.6)	-
Age, years, *Median (IQR)*	67.16 (43–85)	70.0 (51–86)	70.8 (51–86)	68.2 (52–82)	64.1 (43–84)	** < 0.001**
PSA, ng/mL, *Median (IQR)*	8.42 (0.08–86.20)	9.93 (0.5–86.20)	11.08 (1.13–86.20)	7.37 (0.50–20.28)	6.95 (0.08–25.80)	** < 0.001**
Prostate Volume, mL, *Median (IQR)*	53.39 (10–279)	46.99 (10–153)	45.47 (10–153)	50.30 (16–139)	59.41 (11–279)	** < 0.001**
PSA Density, ng/mL^2^, *Median (IQR)*	0.19 (0.002–1.60)	0.26 (0.02–1.60)	0.30 (0.02–1.60)	0.16 (0.02–0.49)	0.13 (0.002–0.98)	** < 0.001**
Experimental PI-RADS						
2, *n (%)*	37 (9.5)	1 (0.3)	0 (0)	1 (0.3)	36 (9.3)	** < 0.001**
3, *n (%)*	80 (20.6)	12 (3.1)	2 (0.5)	10 (2.6)	68 (17.5)	** < 0.001**
3up, *n (%)*	69 (17.7)	17 (4.4)	10 (2.6)	7 (1.8)	52 (13.4)	** < 0.001**
4, *n (%)*	135 (34.7)	104 (26.7)	72 (18.5)	32 (8.2)	31 (8.00)	** < 0.001**
4 + 3p, *n (%)*	204 (52.4)	121 (31.1)	82 (21.1)	39 (10.0)	83 (21.4)	** < 0.001**
5, *n (%)*	68 (17.5)	60 (15.4)	50 (12.8)	10 (2.6)	8 (2.1)	** < 0.001**
Biopsy cores						
Total, *Median (IQR)*	3.6 (1–6)	3.6 (1–6)	3.6 (1–6)	3.6 (2–6)	-	0.819
Positive cores, *Median (IQR)*	2.8 (1–6)	2.74 (1–6)	3.0 (1–6)	2.2 (1–4)	-	** < 0.001**
Positive cores %, *Median (IQR)*	40.8 (3.2–100)	40.8 (3.2–100)	49.6 (6–100)	22.3 (3.2–68.7)	-	** < 0.001**
Positive cores lenght, *Median (IQR)*	1.48 (0.05–6.00)	1.48 (0.05–6.00)	1.80 (0.15–6.00)	0.82 (0.05–4.4)	-	** < 0.001**
ISUP grade (Gleason score), ***n (%)***						
Negative	197 (50.6)	-	-	-	197 (50.6)	-
1 (3 + 3)	61 (15.7)	61 (15.7)	-	61 (15.7)	-	-
2 (3 + 4)	79 (20.3)	79 (20.3)	79 (20.3)	-	-	-
3 (4 + 3)	28 (7.2)	28 (7.2)	28 (7.2)	-	-	-
4 (4 + 4 / 3 + 5 / 5 + 3)	21 (5.4)	21 (5.4)	21 (5.4)	-	-	-
5 (4 + 5 / 5 + 4 / 5 + 5)	3 (0.8)	3 (0.8)	3 (0.8)	-	-	-

^*^ *p* value < 0.05 was considered for statistical significance (bold values within the table), calculated comparing three groups: negative patients, patients with ciPCa and patients with csPCa

*PCa*, prostate cancer; *csPCa*, clinically significant prostate cancer; *ciPCa*, clinically insignificant prostate cancer; *IQR*, interquartile range; *PSA*, prostate-specific antigen; *PI-RADS*, Prostate Imaging—Reporting and Data System; *ISUP*, International Society of Uropathology

A total amount of 284 MRDB was performed, with a mean per-patient number of 3.6 cores taken from each MRI target. One-hundred-ninety-two/389 men (49.4%) were diagnosed with PCa, including 132/389 cases of csPCa (33.9%) and 60/389 cases of ciPCa (15.4%). The remaining 197/389 men (50.6%) were found negative for PCa, with most of them showing inflammatory patterns at histological examination.

### Cancer prevalence according to PI-RADS categories

**Fig. 2:**
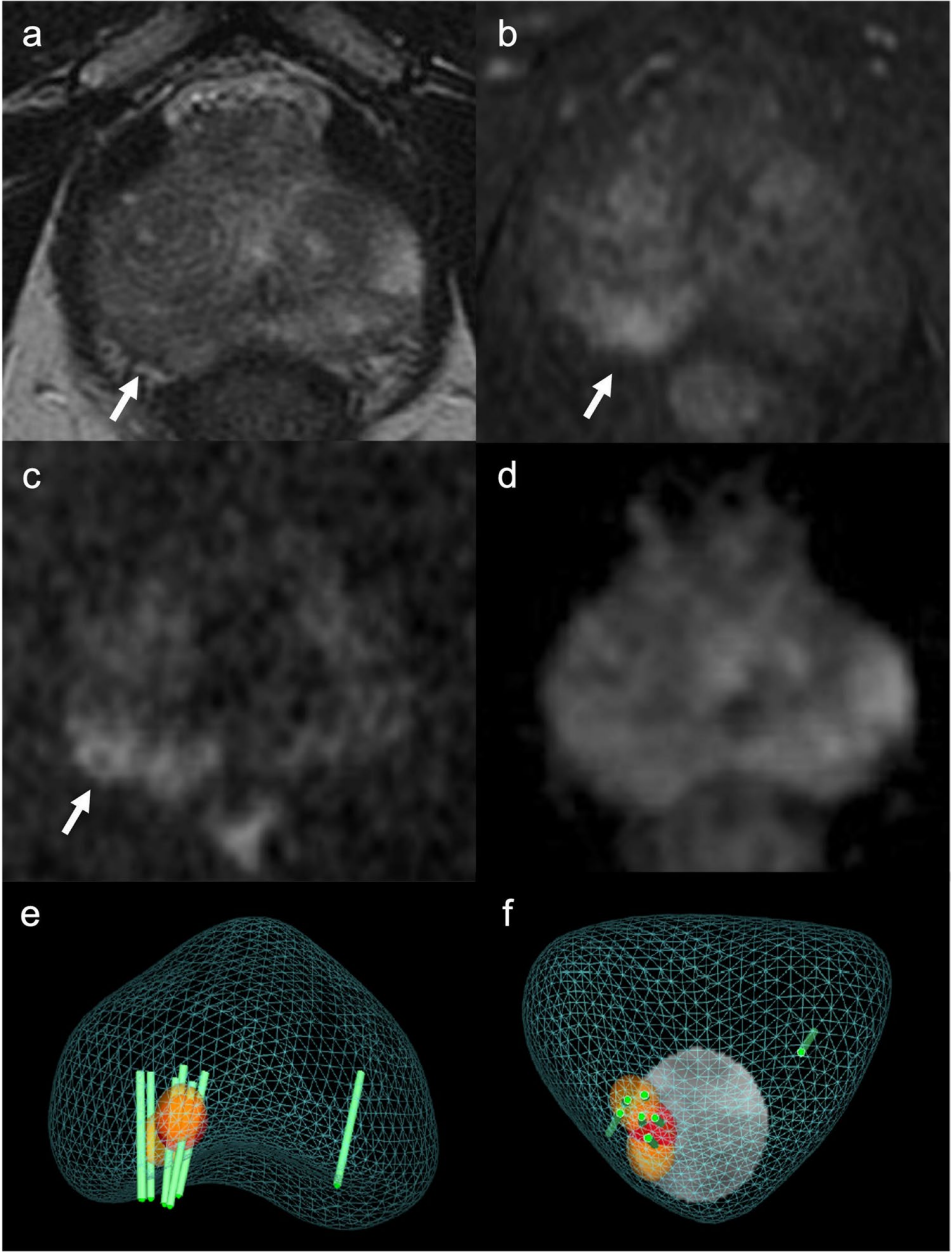
A 73-year-old man with clinical suspicion of prostate cancer (PSA total value of 6.3 ng/mL, PSA density of 0.09 ng/mL^2^, positive family history of prostate cancer). **a** T2WI acquired on the axial plane showing a hypointense nodular lesion on a wider wedge-shaped alteration on the apical-right posterior peripheral zone, with post-contrast enhancement on early DCE images (**b**), with restricted diffusion at b-value 2000 (**c**) and normal ADC value (**d**), classified as PI-RADS 3up. **e**, **f** The lesion was biopsied using MRI-TRUS TBx. Histopathology proved the absence of neoplastic disease, with an inflammatory pattern. PSA, prostate-specific antigen; T2WI, T2-weighted imaging; DCE, dynamic contrast-enhanced; TBx, targeted biopsy

**Fig. 3:**
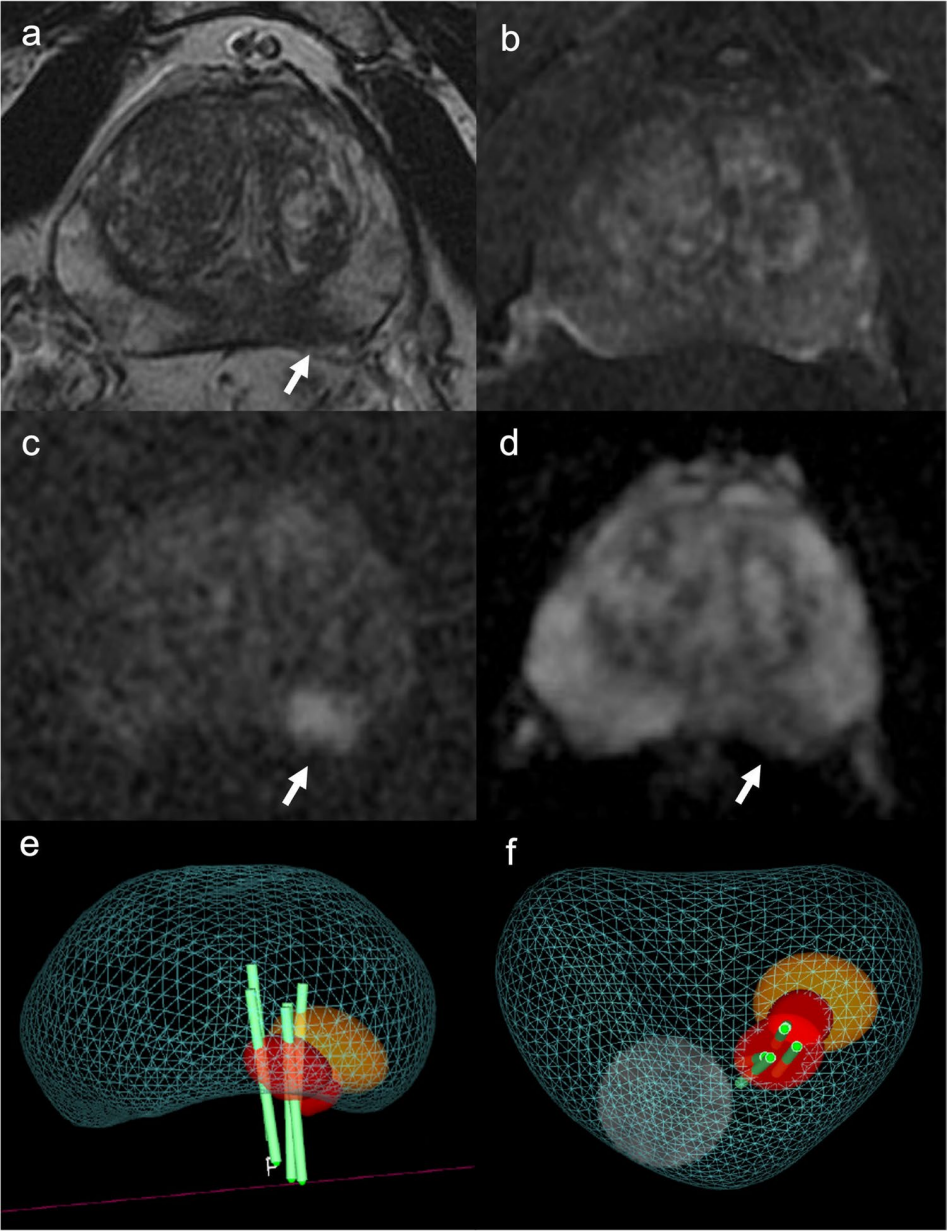
A 62-year-old man with clinical suspicion of prostate cancer (PSA total value of 12.9 ng/mL, PSA density of 0.18 ng/mL^2^). **a** T2WI acquired on the axial plane showing hypointense nodular lesion on the mid-left posterior peripheral zone, with mild post-contrast enhancement on DCE images (**b**), with marked restriction diffusion at b-value 2000 (**c**) and low ADC value (**d**), classified as PI-RADS 4. **e**, **f** The lesions was biopsied using MRI-TRUS TBx. Histopathology confirmed the presence of clinically significant prostate cancer, ISUP 3 (GS 4 + 3). PSA, Prostate-specific antigen; T2WI, T2-weighted imaging; DCE, dynamic contrast-enhanced; TBx, targeted biopsy; ISUP, International Society of Urogenital Pathology; GS, Gleason score

The prevalence of csPCa, ciPCa, and inflammatory patterns in each category of PI-RADS scores is summarized in Table 2. Of note, csPCa prevalence was low in category 3 (2.5%) and 3up (14.5%) (Fig. 2), while we witnessed a drastic increase in PI-RADS 4 (DWI score 4) lesions (53.3%) (Fig. 3), with a statistically significant difference between PI-RADS 3up and PI-RADS 4 both considering PCa prevalence (*p* < 0.001) and csPCa prevalence (*p* < 0.001).

**Table 2:** Prevalence of histologic results among patients with lesions classified with different PI-RADS scores

	PCa	csPCa	ciPCa	Negative	***p*** value*
**3**, *n (prevalence, %)*	12 (15.0)	2 (2.5)	10 (12.5)	68 (85.0)	** < 0.001**
**3up**, *n (prevalence, %)*	17 (24.6)	10 (14.5)	7 (10.1)	52 (75.4)	** < 0.001**
**4**, *n (prevalence, %)*	104 (77.0)	72 (53.3)	32 (23.7)	31 (23.0)	** < 0.001**
**4 + 3up**, *n (prevalence, %)*	121 (78.4)	82 (40.2)	39 (19.1)	83 (40.7)	** < 0.001**
**5**, *n (prevalence, %)*	60 (88.2)	50 (73.5)	10 (14.7)	8 (11.8)	** < 0.001**

^*^ *p* value < 0.05 was considered for statistical significance (bold values within the table), calculated comparing three groups: negative patients, patients with ciPCa, and patients with csPCa

*PI-RADS*, Prostate Imaging—Reporting and Data System; *PCa*, prostate cancer; *csPCa*, clinically significant prostate cancer; *ciPCa*, clinically insignificant prostate cancer

### Diagnostic performance of MRI and PI-RADS assessment

Inter-reader agreement considering the assignment of PI-RADS categories was substantial (*k* = 0.829).

**Fig. 4:**
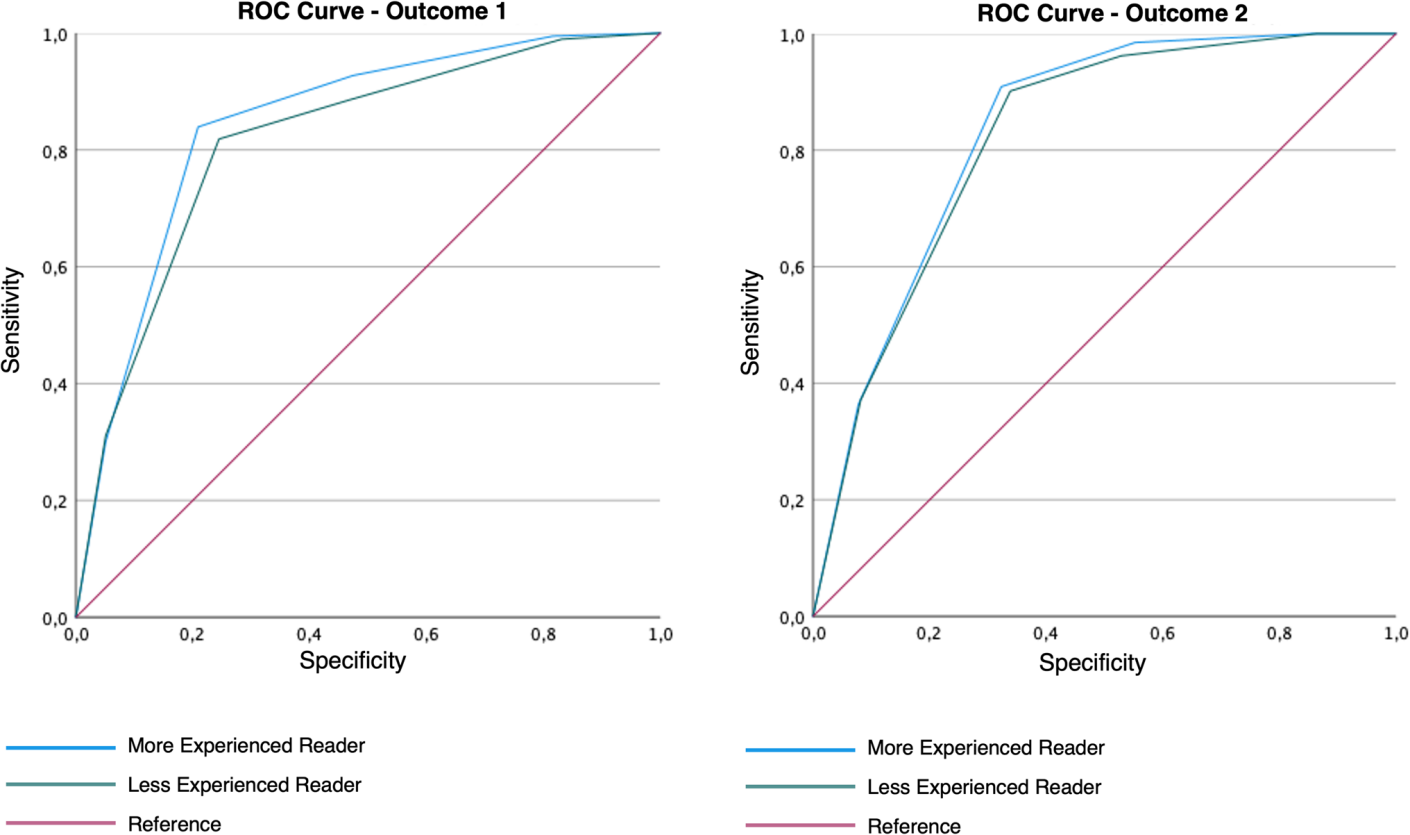
ROC analysis for the performance of MRI and PI-RADS score, in detecting prostate cancer (outcome 1) and clinically significant prostate cancer (outcome 2), for both more and less experienced readers. ROC, receiver operating curve; PI-RADS, Prostate Imaging—Reporting and Data System

MRI showed a sensitivity, specificity, PPV, NPV, and accuracy of 99.0%, 52.6%, 67.2%, 98.1%, and 75.6% versus 100.0%, 40.9%, 46.5%, 100.0%, and 60.9% for outcome 1 versus outcome 2, respectively. The most experienced reader and less experienced reader achieved an area under the curve (AUC) for outcome 1 and outcome 2 of 0.84 (95% CI: 0.80–0.88) and 0.83 (95% CI: 0.79–0.87) versus 0.82 (95% CI: 0.77–0.86), and 0.82 (95% CI: 0.78–0.86), respectively (Table 3; Fig. 4).

**Table 3:** Diagnostic performance and area under the curve of MRI and PI-RADS score with a conventional and adjusted threshold for prostate cancer (outcome 1) and clinically significant prostate cancer (outcome 2) detection

	SENS (%)	SPEC (%)	PPV (%)	NPV (%)	ACC (%)	AUC (95% CI) ^§^
OUTCOME 1						
MRI and PI-RADS score with conventional threshold*	99.0	52.6	67.2	98.1	75.6	0.84 (0.80-–0.88)
MRI and PI-RADS score with adjusted threshold**	92.2	71.4	76.1	90.3	81.7	-
OUTCOME 2						
MRI and PI-RADS score with conventional threshold*	100.0	40.9	46.5	100.0	60.9	0.83 (0.79–0.87)
MRI and PI-RADS score with adjusted threshold**	93.9	57.2	53.0	94.8	69.7	-

^*^ Conventional threshold for considering MRI positive: MRDBs are indicated in case of PI-RADS 5, 4 (DWI score 4), and 3up; PI-RADS 3 only in case of elevated PSA density

^**^ Adjusted threshold for considering MRI positive: MRDBs are indicated in case of PI-RADS 5, 4 (DWI score 4); both 3up and 3 only in case of elevated PSA density

^§^ AUC calculated on the more experienced reader performance

*SENS*, sensitivity; *SPEC*, specificity; *PPV*, positive predictive value; *NPV*, negative predictive value; *ACC*, accuracy; *AUC*, area under the curve; *PI-RADS*, Prostate Imaging—Reporting and Data System

Moreover, considering MRI positive in cases of lesions classified as PI-RADS 5, 4, and both 3 and 3up only when associated with elevated PSA density, MRI showed a sensitivity, specificity, PPV, NPV, and accuracy of 92.2%, 71.4%, 76.1%, 90.3% and 81.7% versus 93.9%, 57.2%, 53.0%, 94.8%, and 69.7% for outcome 1 versus outcome 2, respectively (Table 3).

### Features associated with prostate cancer

Results of the univariable and multivariable analysis are shown in Table 4 and Table 5. The multivariable logistic regression model, for what concerns both outcomes 1 and 2, showed that the variables independently correlating with PCa were age (both *p* < 0.001), PSA density (*p* = 0.03 and < 0.001 respectively), DWI as best sequence (both *p* < 0.001), conventional PI-RADS score (*p* = 0.007 and = 0.04 respectively) and adjusted PI-RADS score (*p* < 0.001 and 0.03).

**Table 4:** Univariable and multivariable regression analysis assessing the correlation among clinical and radiological factors with prostate cancer

OUTCOME 1	Evidence of Tumor *n* (%) – 389		p*	ODDS RATIO*	p**	ODDS ratio**
	Not	Yes				
Age *(mean)*			** < 0.001**	0.31 (0.21–0.40)	** < 0.001**	0.14 (0.06–0.22)
< 70	148	87				
≥ 70	49	105				
Family history			0.41	0.63 (1.20–1.97)	–	–
NO	189	187				
YES	8	5				
PSA *(ng/mL)*						
< 10	159	135	**0.02**	0.57 (0.35–0.91)	0.46	0.04 (0.01–0.41)
> 10, < 20	36	41	0.48	1.21 (0.74–2.00)	–	–
> 20	2	16	** < 0.001**	0.17 (0.03–0.34)	0.51	0.12 (0.02–0.31)
PSA density *(ng/mL*^*2*^*)*			** < 0.001**	0.35 (0.25–0.43)	**0.03**	0.10 (0.09–0.11)
< 0.15	150	81				
≥ 0.15	47	111				
Number of foci (n)			0.24	0.07 (0.03–0.16)	–	–
1	98	83				
> 1	99	109				
Best sequence			** < 0.001**	0.55 (0.46–0.63)	** < 0.001**	0.22 (0.13–0.32)
DCE	148	38				
DWI/ADC	49	154				
Conventional PI-RADS score §			** < 0.001**	0.65 (0.56–0.74)	**0.007**	0.17 (0.05–0.30)
Adjusted PI-RADS score §§			** < 0.001**	0.66 (0.59–0.74)	** < 0.001**	0.32 (0.19–0.45)

^*^ Univariate analysis; *p* value < 0.05 was considered for statistical significance (bold values within the table)

^**^ Multivariable analysis; *p* value < 0.05 was considered for statistical significance (bold values within the table)

^§^ PI-RADS with the conventional threshold to auction biopsy (PI-RADS 3 associated with PSA density ≥ 0.15 ng/mL/mL) assigned by an expert reader

^§§^ PI-RADS with the adjusted threshold to auction biopsy (both PI-RADS 3 and 3up associated with PSA density ≥ 0.15 ng/mL/mL) assigned by an expert reader

*PSA*, prostate-specific antigen; *PI-RADS*, Prostate Imaging—Reporting and Data System

**Table 5:** Univariable and multivariable regression analysis assessing the correlation among clinical and radiological factors with clinically significant prostate cancer

OUTCOME 2	Evidence of csPCa *n* (%) – 389		p*	Odds ratio*	p**	Odds ratio**
	Not	Yes				
Age *(mean)*			** < 0.001**	0.29 (0.19–0.38)	** < 0.001**	0.15 (0.06–0.23)
< 70	182	53				
≥ 70	75	79				
Family history			0.40	0.57 (0.15–2.12)	–	–
NO	247	129				
YES	10	3				
**PSA ***(ng/mL)*						
< 10	208	86	** < 0.001**	0.19 (0.03–0.28)	0.95	1.16 (0.58–2.33)
> 10, < 20	45	32	0.11	0.41 (0.09–0.87)	–	–
> 20	4	14	** < 0.001**	0.46 (0.24–0.68)	0.11	0.21 (0.04–0.64)
PSA density *(ng/mL*^*2*^*)*			** < 0.001**	0.34 (0.25–0.43)	** < 0.001**	0.21 (0.07–0.25)
< 0,15	185	46				
≥ 0,15	72	86				
Number of foci (n)			0.58	0.89 (0.58–1.35)	–	–
1	117	64				
> 1	140	68				
Best sequence			** < 0.001**	0.42 (0.34–0.51)	** < 0.001**	0.21 (0.12–0.30)
DCE	164	22				
DWI/ADC	93	110				
Conventional PI-RADS score §			** < 0.001**	0.46 (0.37–0.56)	**0.04**	0.14 (0.02–0.28)
Adjusted PI-RADS score §§			** < 0.001**	0.48 (0.39–0.56)	**0.03**	0.32 (0.19–0.45)

^*^ Univariate analysis; *p* value < 0.05 was considered for statistical significance (bold values within the table)

^**^ Multivariable analysis; *p* value < 0.05 was considered for statistical significance (bold values within the table)

^§^ PI-RADS with the conventional threshold to auction biopsy (PI-RADS 3 associated with PSA density ≥ 0.15 ng/mL/mL) assigned by an expert reader

^§§^ PI-RADS with an adjusted threshold to auction biopsy (both PI-RADS 3 and 3up associated with PSA density ≥ 0.15 ng/mL/mL) assigned by an expert reader

*PSA*, prostate-specific antigen; *PI-RADS*, Prostate Imaging—Reporting and Data System

## Discussion

In this study, we applied an adjusted version of the PI-RADS scoring system, considering prostate peripheral zone lesions. We differentiated lesions classified as PI-RADS 4 (with DWI score 4) from those upgraded from PI-RADS 3 (DWI score 3) because of positive DCE and identified as PI-RADS 3up. Moreover, we considered a different threshold for defining MRI positive and setting indication to prostate biopsy: both PI-RADS 3 and PI-RADS 3up are associated with increased PSA density. Our analysis resulted in a reduction of MRI false positives and a significant increase in csPCa prevalence among patients with PI-RADS 4 lesions. Furthermore, in our analysis, we witnessed a marked improvement of PPV when PI-RADS 3up lesions are directed to MRDB only in case of association with elevated PSA density. PPV is currently still low and high-variable among centers [11]; this was probably because PI-RADS 4, including lesions upgraded due to a positive DCE, is the second most frequent source of false positive assignments after PI-RADS 3 [24].

These results strengthened the diagnostic power of PI-RADS 4 categorization in the detection of csPCa, with the potential effect of reducing the excess of false positives, already widely described in the literature [15]. This analysis could have a potentially positive effect on biopsy planning, still maintaining the high sensitivity and NPV of MRI.

The secondary objective was to define the clinical and radiological features independently correlating with the presence of PCa and of csPCa, to profile patients at higher risk. The logistic regression models demonstrated how age, PSA Density ≥ 0.15, a clearer definition of the lesion on DWI (compared to DCE), and the PI-RADS score show independent correlation with csPCa. In this sense, when considering PI-RADS 3up as PI-RADS 3 and no longer as PI-RADS 4, 2.6% of csPCa would have been missed if not considering additional data. These results may suggest that clinical-radiological data, if included in nomograms, could support a risk-based stratification of PI-RADS 3 and 3up lesions.

Considering what we found, a new subcategorization of PI-RADS 3 lesions could be proposed: (i) PI-RADS 3B consisting of PI-RADS 3 and 3up findings requiring to be directed to biopsy according to clinical data; and (ii) PI-RADS 3FU, consisting of PI-RADS 3 and 3up findings that should be directed to a follow-up MRI.

Expectedly, DCE did not independently correlate with csPCa detection, which reflects its impact on the number of false positive MRI findings. Indeed, as already proved by other studies, DWI plays a key role in the diagnostic workup of PCa and the applicability of non-contrast MRI in the clinical practice is becoming more and more appealing and necessary, given the requested high burden of MR [25], and also given its comparable diagnostic accuracy in the detection of csPCa [26–28]. However, as properly assessed by Belue et al. [29], this is still a controversial topic, since data are limited; studies found in literature often lack the stratification of findings according to prostate cancer grade group per PI-RADS score, the image quality assessment, and readers’ experience [30]. The present study considered all these aspects, proving the relevance of DWI and the secondary role of DCE, as an additional sequence to upgrade equivocal lesions. Our results suggest limiting the DCE’s implication in the scoring assessment and support the use of non-contrast MRI in selected cases, especially in high-volume referral centers, where expert radiologists interpret the images. Moreover, these results are in line with the results of the meta-analysis by Zeng et al. [31], who found that DCE’s role is limited in case of equivocal lesions and expressed concerns on the need of a more suitable method to improve the detection of csPCa. In this sense, the adjusted PI-RADS threshold to direct patients to MRDB proposed in this study could represent the answer to their conclusions. Nonetheless, DCE could still be valuable in cases where DWI and/or T2WI do not reach a sufficiently good quality level [29, 32]; however, we did not investigate it in our study.

Limitations need to be acknowledged: first, the single-center study was conducted in a high-volume referral center with both readers being experienced; second, low-quality exams were excluded from the analysis. These might hamper the reproducibility of the results. Finally, we acknowledge that proposing in a retrospective fashion a new threshold for defining an MRI finding as positive could cause a selection bias in future investigations, but we believe that this study could represent a first experience in this scenario, setting the base for future research and randomized trials.

In conclusion, we can affirm that the upgrade of lesions with DWI Score 3 to PI-RADS 4 given a positive DCE MRI does not provide a positive impact on the overall diagnostic performance of MRI for the detection of clinically significant prostate cancer, leading to a reduced cancer prevalence yield. Moreover, these results might boost the applicability of non-contrast MRI for selected populations and centers. Finally, a new subcategorization of PI-RADS 3 scoring could be proposed, consisting of PI-RADS 3 and 3up findings, and divided into: PI-RADS 3B requiring to be directed to biopsy according to clinical data, and PI-RADS 3FU, to be followed-up with MRI.

## Supplementary Information

Below is the link to the electronic supplementary material.

Supplementary file1 (PDF 93 KB)

## Abbreviations

ADC: Apparent diffusion coefficient
AUC: Area under the curve
ciPCa: Clinically insignificant prostate cancer
csPCa: Clinically significant prostate cancer
DCE: Dynamic contras-enhanced
DRE: Digital rectal examination
DWI: Diffusion-weighted imaging
ESUI: EAU (European Association of Urology) Section of Urological Imaging
ESUR: European Society of Urogenital Radiology
IQR: Interquartile range
ISUP: International Society of Urological Pathology
MRDB: MRI-directed biopsies
MRI: Magnetic resonance imaging
NPV: Negative predictive value
PCa: Prostate cancer
PI-QUAL: Prostate Imaging Quality
PI-RADS 3up: PI-RADS 3 upgraded to PI-RADS 4
PI-RADS: Prostate Imaging—Reporting and Data System
PPV: Positive predictive value
PSA: Prostate-specific antigen
PSAD: Prostate-specific antigen density
PZ: Peripheral zone
ROC: Receiver operating characteristic

## Declarations

### Guarantor

The scientific guarantor of this publication is Valeria Panebianco.

### Statistics and biometry

No complex statistical methods were necessary for this paper.

### Study subjects or cohorts overlap

N.A.

### Methodology

• Prospective
