# Supplementary material for: Low cancer yield in PI-RADS 3 upgraded to 4 by dynamic contrast-enhanced MRI: is it time to reconsider scoring categorization?
Source: Eur Radiol. 2023 Apr 13;33(8):5828–39. doi: 10.1007/s00330-023-09605-0 (PMC10326099; doi:10.1007/s00330-023-09605-0)
Supplement: Supplementary file 1 — Supplementary file1 (PDF 93 KB) [file 330_2023_9605_MOESM1_ESM.pdf]

ELECTRONIC SUPPLEMENTARY MATERIAL

Low cancer yield in PI-RADS 3 upgraded to 4 by Dynamic contrast-enhanced MRI: is it time to reconsider scoring categorization?

Supplementary Table 1. DWI scoring system according to PI-RADS v2.1 recommendations [17]. DWI, diffusion weighted imaging; ADC, apparent diffusion coefficient; PI-RADS, Prostate Imaging - Reporting and Data System.

DWI SCORE	DEFINITION
**1**	No evidence of alterations on high b-value DWI and on ADC map
**2**	Linear/wedge shaped hyperintensity on high b-value DWI and/or linear/wedge shaped hypointensity on ADC map
**3**	Focal mild hyperintensisty on high b-value DWI and/or focal mild hypointensity on ADC map; marked hyperintensity on high b-value DWI or marked hypointensity on ADC, but not both
**4**	Focal marked hyperintensity on high b-value DWI and marked hypontensity on ADC (<1.5cm)
**5**	Same characteristics as 4 but ≥1.5cm or evidence of extraprostatic extension

Eur Radiol (2023) Messina E, Pecoraro M, Laschena L, et al.

Supplementary Table 2. Summary of the MR Acquisition Parameters.
TE, Time of echo; TR, Repetition time.

Manufacturer	**SIEMENS**	**GENERAL ELECTRIC**
Magnet (Tesla)	3	3
Manufacture year	2021	2010
Year of last update	2022	2020
**T2-WI**		
TE (msec)	134	134
TR (msec)	6000	6000
In-plane resolution (mm x mm)	0,4 x 0,4	0,562 x 0,804
Field of view	18x18	18x18
Slice thickness (mm)	3	3
Matrix size	384 x 384	384 x 384
**DWI**		
TE (msec)	75	75
TR (msec)	4300	4300
In-plane resolution (mm x mm)	1,8 x 1,8	1,8 x 1,8
Field of view	18x18	18x18
Slice thickness (mm)	3	3
Multiple b values (sec/mm^2)	100-800-1000	100-800-1000
High b value (sec/mm^2)	2000	2000
Matrix size	90 x 90	90 x 90
**DCE**		
TE (msec)	1	1
TR (msec)	3	3
In-plane resolution (mm x mm)	1,1 x 1,2	1,1 x 1,2
Field of view	18x18	18x18
Slice thickness (mm)	3	3
Fat suppression / subtraction	No	No
Temporal resolution (seconds)	6	6
Matrix size	160 x 140	160 x 140
